# Key drivers involved in the telemonitoring of covid-19 for self-health management: an exploratory factor analysis

**DOI:** 10.1186/s12913-022-07828-3

**Published:** 2022-04-19

**Authors:** Letizia Lo Presti, Mario Testa, Giulio Maggiore, Vittoria Marino

**Affiliations:** 1 Department of Law and Economics, University of Rome “Unitelma Sapienza”, Rome, Italy; 2 Department of Management and Innovation Systems, University of Salerno, Fisciano, SA Italy; 3 Department of Law, Economics, Management and Quantitative Methods, University of Sannio, Benevento, Italy

**Keywords:** Health engagement platform, Cognitive engagement, COVID-19, Satisfaction, Telemonitoring, Self-health engagement, Health digital platform

## Abstract

**Background:**

The recent COVID-19 pandemic and the shortage of general practitioners has determined a strong pressure on the Italian health system. This critical issue highlighted the fundamental support of e-health services not only to lighten the workload of doctors, but also to offer patients a health service tailored to real needs. Therefore, the digital engagement platforms represent a valid aid, as they reconcile the efficiency needs of the healthcare system with the benefits for the patients involved. In this perspective, little is known about the main factors associated with use of telemonitoring platforms and their effectiveness. This paper investigates the critical success factors of telemonitoring platforms during COVID-19 in order to understand the mechanisms underlying patient participation with the health engagement platforms.

**Methods:**

An exploratory factor analysis was used to explain the main dimensions of patient participation in the COVID-19 telemonitoring. A sample of 119 patients with a suspected or confirmed infection was used in the investigation. Moreover, an analysis of variance was calculated to identify the differences between three types of patients (infected, uninfected, with suspected infection) and verify the effectiveness of the platform.

**Main Findings:**

There are six main factors underlying the use of the COVID-19 telemonitoring platform. “Self-Health Engagement” emerges as a novel factor. Moreover, compared to other platforms, cognitive engagement is a crucial trigger for effective telemonitoring.

**Discussion:**

By identifying the main triggers involved in the use of health engagement platforms, we can improve the satisfaction of telemonitoring services for appropriate health-crisis management. Furthermore, the COVID-19 telemonitoring platform appears to improve health management for both patients and health care providers as it provides the patient with the necessary tools for Self-Health Management (SHM), as well as helping to enrich the literature on health care.

**Conclusion:**

A new construct emerges in the study of digital telemonitoring platforms: “health self-engagement”, that is, an engagement based on self-care that demonstrates the decisive role assumed by both digital technology and patient participation in self-management.

**Supplementary Information:**

The online version contains supplementary material available at 10.1186/s12913-022-07828-3.

## Background

Digital technology presents enormous opportunities for improvement in terms of the efficiency and effectiveness of health systems. E-health already offers digital solutions (telemedicine, telemonitoring, digital medical records, online health community, etc.) that guarantee a better quality of care at a more sustainable cost [1]. The diffusion of COVID-19 has led to a worldwide push for the transition of these solutions [2], allowing doctors, patients and the community in general to protect themselves from the risk of exposure and contagion. Furthermore, in Italy, according to the Italian Federation of General Practitioners (GP), in the next 5 years, almost 15,000 family doctors will stop practising, with the risk of leaving about 14 million Italians without a general practitioner [3].

Having overcome the regulatory barriers of the individual states that are now moving forward towards the recognition of telemedicine as the main tool for treatment even during pandemic crises, the factors that facilitate the doctor-patient relationship through digital platforms still need to be investigated in greater depth. The results of many studies, in fact, have demonstrated the importance of the patient's active role in the effectiveness of care [4–6] and the radical evolution of an approach that is more and more patient-centred [7, 8].

In particular, self-management in the health sector, i.e. the transfer to the patient of skills and activities normally falling within the competencies of the doctor [9], has shown its effectiveness especially in those cases that manifest urgency or the need for hospitalization [10].

The propensity to use telemedicine seems to increase as the relationship with the doctor, or in any case with the service provider, becomes more consolidated [11]. Other studies have investigated the need to ensure the security, availability and sustainability of this kind of technology over the long term [10]. Meskó et al. [12] highlight in their research how misleading it can be to focus attention exclusively on the technological component and not on the human one. The authors stressed that patients who had not been previously engaged or informed either on how to use digital tools or on their state of health, did not experience any significant improvement in their health status. More specifically, other studies have shown that the success of performance and care through digital platforms depends on collaboration, empathy and shared decision-making [13]. In this regard, it appears useful to summarize the main categorizations that the literature has proposed to classify the mechanisms that are triggered when the patient interacts with a digital health platform in this ambit (Table 1) and the main benefits and problems encountered in the literature regarding the health sector.

**Table 1:** – The main theoretic constructs of reference

Constructs	Definition	Advantages/concerns in e-health platforms
Consumer Engagement	“Customer engagement (CE) is a psychological state that occurs by virtue of interactive, co-creative customer experiences with a focal agent/object (e.g., a brand) in focal service relationships.” [14:260] “Consumer engagement is a multidimensional concept comprising cognitive, emotional, and/or behavioral dimensions, and plays a central role in the process of relational exchange where other relational concepts are engagement antecedents and/or consequences in iterative engagement processes within the brand community [15:107]	Efficiency and effectiveness**—**Patient engagement guarantees efficiency and effectiveness in the health system and a reduction in health costs because it is anchored on patient participation [6, 16–19]
Consumer Satisfaction	“Consumer satisfaction is a response (emotional or cognitive); 2) the response pertains to a particular focus (expectations, product, consumption experience, etc.); and 3) the response occurs at a particular time (after consumption, after choice, based on accumulated experience, etc.) [20] “Satisfaction is defined as a global evaluation or feeling state” [21:256]	Cooperation—The higher the level of satisfaction the more the patient will follow the treatment and cooperate with the doctors and health workers [22, 23]
Perceived benefit	“Perceived benefit refers to the perceived likelihood that taking a recommended course of action will lead to a positive outcome, such as reduced risk or reduced worry” [24:36] “Benefits refer to the expected or experienced positive consequences of [a given behavior]” [25:50] “Perceived benefits construct is […] defined as an individual’s belief that specific positive outcomes will result from a specific behavior” [26:88]	Intention to use—The benefits perceived by the patient are inversely proportional to the difficulty perceived in the use of the technology [27]. The expected benefits have an effect on the intention to use the digital health platform [28]
Perceived Technological Risk	“(…) is commonly thought of as felt uncertainty regarding possible negative consequences of using a product or service” [29:453] “(…) the potential for loss in the pursuit of a desired outcome of using an e-service” [29:454]	Reliability of health services—The level of security of the patient’s clinical data and their correct storage on the web increases the level of reliability of the health services provided through the digital health platform [30]
Effort Expectancy	“(…) is defined as the degree of ease associated with the use of the system” [31:509]	Intention to use—The patient’s effort expectancy affects the intention to use a digital health service [28]
Perceived Usefulness	“The prospective user’s subjective probability that using a specific application system will increase his or her job performance within an organizational context’’ [32:985] “(…) the degree to which a person believes that using a particular system would enhance his or her job performance” [33:320]	Positive attitude of patients—The perception of usefulness of digital health services favorably predisposes the patients and facilitates the doctor’s decision-making process (medical decision making) [34]
Perceived Ease of Use	“(…) the degree to which an innovation is perceived as being difficult to use [35:195] “(…) the degree to which a person believes that using a particular system would be free of effort” [33:320]	Collaboration—The perceived ease of use of digital health services encourages the patient to continue using the platform [28] and guarantees a greater predisposition in the patient to collaborate and follow the treatment program [36]

Once again, satisfaction is closely linked to engagement in this context. In fact, some studies have shown that satisfaction is significantly influenced by customer engagement, in particular by the cognitive and emotional dimension, during the use of digital technology. A high level of engagement during interaction through technology is a valid antecedent for satisfaction, positive word of mouth and the consolidation of long-term relationships [37, 38].

With particular reference to the patients' perception of benefits and risks in using the technology, Win et al. (2016) [26] grouped perceived benefits into health perceived benefits and perceived benefits in a social context. Each of the two categories has different dimensions that are subject to specific measurements in different studies. The dimensions investigated for the perceived health benefits refer to improved health outcome [39], patient awareness [40], health self-management [41], adherence to cure and treatment [42]. In the same way, much research has focused on evaluating the users’ perception of risk in the use of technology by its. Drawing on the Theory of Perceived Risk [43–45], some aspects of risk have been made operational, empirically tested and above all integrated into the Technology Acceptance Model [29]. The results show that the negative perception of risk associated with the use of digital platforms is mitigated by the ease of use of the tool and its ability to improve the desired results and performances. Furthermore, even if users perceive the usefulness of the technological tool, they may at the same time see it as too difficult to use. Therefore the achievable benefits in terms of performance are negatively offset by the effort made to use it. Many studies have shown that effort expectancy affects the intention to adopt a new technology [28, 46–48] even if this is more noticeable in the early stages of its use [31] and dependent on the personal experience of the individual [44].

In light of the foregoing, the present work intends to investigate the use of digital platforms for the management of patients affected by COVID-19 whose different variants modify the clinical manifestations and increase transmissibility, morbidity and mortality [49, 50]. Above all, the factors that influence the doctor-patient relationship will be analyzed in order to improve both the engagement and satisfaction of the doctor and the self-management of health by the patient, with considerable advantages for the entire health system.

## Methods

### Data collection procedure

To investigate the mechanisms underlying patient participation on digital health platforms, the opinions and experiences of patients who have used digital health platforms for monitoring COVID-19 were collected between June 2020 and November 2020. Google Forms was used to create the online survey and once the link to the online survey was generated, the Italian platform of Paginemediche.it, which hosts on its portal a home telemonitoring service for patients with a suspected or confirmed infection of SARS CoV-2, was used for its administration. The home telemonitoring service activated in collaboration with the Italian Ministry of Health, allows doctors and patients to interact and share data remotely and in complete safety. The platform, created by Paginemediche.it in collaboration with family doctors, helps to keep symptoms under control and to intervene promptly. The patient with suspected or confirmed SARS CoV-2 infection can activate the telemonitoring service, monitor his symptoms and parameters and send them to his doctor. The patient can invite his family doctor to activate the service so as to monitor his state of health day by day.

The questionnaire was sent by e-mail to all those patients who, because of the health emergency, had registered for this specific service on the Paginemediche.it portal. A total of 500 emails with the invitation to participate in the survey was sent between June 2020 and November 2020. The patients, registered on the Paginemediche.it platform, were informed of the objective of the research and at the end of the survey period we collected 119 questionnaires out of a total of 500 invitations. After an introductory section aimed at categorizing the type of patient with respect to a suspected or ascertained SARS CoV-2 infection, the online questionnaire presented a section on the level of engagement and satisfaction of the service, on its usefulness and finally on its perceived benefits. The questionnaire ended with a section to gather information on the demographic characteristics of the patient. Anonymity was guaranteed during the data collection. In fact, the survey was carried out in compliance with the patient's privacy and the data was processed in an aggregate form and without allowing any recognition of the patient.

Measurement development.

The tools used to measure the constructs in this study were adapted from previous studies to ensure the validity of the content. Detailed information on the constructs and sources is given in Table 2. Answers were given on a seven-point Likert scale (from 1 strongly disagree, to 7 strongly agree). A reliability analysis was carried out on the data by calculating the Item-to-Total Correlation (ITC) index and Cronbach's α for each scale was included in the model.

**Table 2:** Descriptive statistics of constructs

Constructs	Authors	N. items		μ	DS	Variance	Min index	Max index	Alpha di Cronbach
Cognitive engagement	Hollebeek et al. (2014) [51]		3	4.65	1.97	3.88	4.52	4.74	0.93
Emotional engagement	Hollebeek et al. (2014) [51]		3	4.95	1.73	3.00	4.57	5.39	0.91
Behavioural engagement	Hollebeek et al. (2014) [51]		3	4.29	1.79	3.22	3.66	4.66	0.81
Satisfaction	Wang et al., 2004 [52]		3	5.49	1.43	2.07	5.42	5.60	0.90
Perceived benefit	Win et al. (2016) [26]		4	4.31	1.82	3.31	4.05	4.67	0.92
Perceived Technological risk	Chen and Aklikokou (2020) [53]		3	2.98	1.61	2.61	2.93	3.03	0.83
Effort Expectancy	Venkatesh et al. (2012) [54]		4	3.34	1.60	2.56	3.09	3.52	0.81
Perceived usefulness	Davis et al. (1989) [33]		4	5.29	1.55	2.41	5.11	5.56	0.93
Perceived ease of use	Davis et al. (1989) [33]		4	5.53	1.39	1.94	4.95	5.79	0.90

At the end of this phase, all measurement scales had Cronbach’s α > 0.80 and all indicators showed an ITC of 0.43. At the end of this phase, as reported in Table 2, all the measured scales were close to or above the recommended value for Cronbach's α (⩾0.7) and for the Composite Reliability Coefficient (CRC) (⩾0.6). We then calculated the overall indices using the mean calculation for each construct. Table 2 provides information on the means of each construct under investigation. The indices show that all constructs have a value almost always higher than the mean value of the Likert scale (mean = 3.5) and with a low/moderate perception of risk (mean = 2.98). Overall, patients are satisfied with the COVID-19 telemonitoring platform (μ =  > 5.49). Customers feel involved, especially under cognitive and emotional profiles (μ = 4.65 and μ = 4.95). Moreover, the platform is perceived as valuable and easy to use (μ = 5.29 and μ = 5.53).

### Statistical analyses

In order to identify the main factors that can explain the mechanisms underlying patient participation with COVID-19 telemonitoring platforms, we conducted an exploratory factor analysis (Maximum likelihood method, Promax rotation criterion) providing an insight into the number and the factors that can best explain the telemonitoring service from the patient's point of view. The purpose of the factor analysis is to identify some latent variables (factors) capable of highlighting the links, interrelationships and dependencies between the observed statistical variables [55]. The “Kaiser rule” was used to extract the components. This type of survey methodology is the most appropriate for an exploratory phase of the phenomenon [55].

Finally, to assess whether there are differences between the factors detected through the exploratory factor analysis and the type of patient (with suspected, ascertained or free from the COVID-19 infection) an ANOVA test was conducted based on the scores of the factors obtained with the exploratory factor analysis and comparing the means of the three groups. From the comparison it was possible to establish whether the three groups were significantly different in their activating mechanisms on the platform and which factors determine the main differences.

## Results

### Descriptive analysis

The descriptive statistics of the sample are available in Table 3. Out of the 119 participants, 62.2% are women while 37.8% are men.

**Table 3:** Descriptive statistics of respondent characteristics

Variable	N	Percentage
**Age**		
Below 25	2	1.7
25 – 35	22	18.5
36 – 45	22	18.5
46 – 55	39	32.8
56 – 65	20	16.8
Above 65	14	11.8
**Gender**		
F	74	62.2
M	45	37.8
**Profession**		
Student	2	1.7
Worker	86	72.3
Unemployed	9	7.6
Retired	18	15.1
Housewife	4	3.4
**Are you a patient with a suspected or ascertained COVID-19 infection?**		
No	40	33.6
Yes	64	53.8
Perhaps	15	12.6
**Why are you using the home telemonitoring service ‘paginemediche.it’**		
For easy access to health information that could help me to prevent illnesses	24	20.2
For speedy access to health services	6	5.0
To have access to the treatment needed for the cure	10	8.4
To monitor my health status post-COVID-19	44	37.0
Other	35	29.4
**How long have you been using the COVID-19 home telemonitoring platform ‘paginemediche’?**		
Less than a week	0	0
A week	17	14.3
Two weeks	21	17.6
Three weeks	15	12.6
A month	16	13.4
More than a month	50	42

Almost 33% are between 46 and 55 years old, all the other participants are equally distributed except for a low representation of young people under the age of 25 (only about 2% of the participants). However, this reflects the socio-demographic characteristics of Italian patients who were affected by the SARS CoV-2 infection during the survey period [56]. Out of the 119 participants in the study, 72% are employed while 15% are retired. Half of the patients said they had COVID-19 (53%) while almost 34% said they did not have the virus; finally, 12.6% stated that they had probably contracted the virus and were awaiting test results. Most patients had been using the telemonitoring service for more than a month (32%) to monitor their health. Among other reasons, the patients stated that they had registered on the platform on the recommendation of their family doctor in order to monitor the data on their health status and keep the family doctor updated on the evolution of the disease (other, 29.4%). This is consistent with Welch et al. (2017) according to whom it is possible that patients will choose to use telemedicine if recommended by their general practitioners (GP) and with whom they have a consolidated relationship.

Factors involved in the telemonitoring COVID-19 for self-health management.

The results of the exploratory factor analysis generated 6 factors. Table 4 shows the factorial model and the loadings (loadings below | 0.40 | are not shown). Both the Bartlett sphericity test, significant result (< 0.001, df = 465), and the KMO sample adequacy measure of 0.881 (> 0.50) confirmed the appropriateness of developing a factor analysis . All the variables showed a commonality equal to or greater than 0.50, showing a good overall significance of the analysis. Cronbach's α reliability coefficient for the single factors was satisfactory (1st factor: 0.95; 2nd factor: 0.88; 3rd factor: 0.90; 4th factor: 0.90; 5th factor: 0.93; 6th factor: 0.93).

**Table 4:** Factors underlying the telemonitoring platform for self-health management

Factor						
	1	2	3	4	5	6
	Health self-engagement	Technological risk	Perceived ease of use	Satisfaction	Patient cognitive engagement	Perceived usefulness
PB1	.871					
ENGe2	.848					
PB3	.809					
ENGe3	.799					
PB4	.764					
ENGb2	.757					
ENGb3	.740					
PB2	.739					
ENGb1	.722					
ENGe1	.665					
EE2		.859				
PTR2		.843				
PTR1		.815				
EE4		.772				
PTR3		.672				
EE3		.600				
EE1	.416	.493				
PEU2			.963			
PEU1			.913			
PEU4			.819			
PEU3			.552			
SAT2				.814		
SAT1				.783		
SAT3				.586		
ENGc2					.875	
ENGc1					.757	
ENGc3					.638	
PU2						.837
PU1						.828
PU3						.560
PU4						.530
Eigenvalue	12.285	3.797	1.792	2.968	.959	.828
Percent of variance	39.660	12.249	5.781	9.573	3.095	2.670
Cumulative percent of variance	39.660	51.909	57.690	67.263	70.358	73.028

Extraction Method: Maximum Likelihood. Rotation Method: Promax with Kaiser Normalization. *ENGc*  Cognitive engagement, *ENGe*  Emotional engagement, *ENGb*  Behavioural engagement, *SAT*  Satisfaction, *PB*  Perceived benefit, *PTR*  Perceived Technological risk, *EE*  Effort Expectancy, *PU*  Perceived usefulness, *PEU*  Perceived ease of use

Finally, the explained total variance was 73%. As you can see in Table 4, in the exploratory factor analysis a cross loading can be found in item EE1: "Adaptation to the telemonitoring service requires a significant commitment" (see also Appendix A which reports the items) which falls both in the "health self-engagement" factor and in the "technological risk" factor. This result is due to the nature of the item which refers to the patient's commitment during the use of the platform (both in emotional terms and in relation to their level of technological skills).

The results shown below identify the main factors that intervene during the patient's interaction with the COVID-19 telemonitoring platform. These results help to understand the mechanisms underlying the use of digital health platforms and their effectiveness, especially in times of pandemics.

- 1. Health self-engagement: The first dimension (which explains 39.6% of the variance of the phenomenon) can be briefly defined as health self-engagement, as it consists of items relating to aspects concerning self-care in terms of benefits perceived by the use of the telemonitoring platform. Any improvement in the state of health passes first through the emotional and behavioural engagement of the patient, pushing him to provide for his psycho-physical well-being through the support provided by digital technology. This is also consistent with the research conducted up on patient engagement in the healthcare sector [57] which identifies the emotional component, and especially empathy during the interaction in a patient social network system, as an important factor affecting patient engagement and consequently the intention to use the platform [58]. Therefore, the emotional and behavioral dimensions, as well as the perceived benefits contribute to rendering effective the management of one's own health. Health self-engagement is an unprecedented factor in the panorama of the factors that are activated on the digital health platforms and is the main contribution of this paper.
- 2. Technological risk.The second dimension (which explains 12.24% of the variance of the phenomenon) can be briefly defined as the technological risk, as it is composed of constructs related to the risk and difficulty perceived by the patient in using a new technological tool. Factors related to privacy, security or changing one's habits are aspects that can interfere with the use of the healthcare platform and therefore discourage its adoption. The combination of technological risk construct and effort expectancy construct in the same factor is consistent with the literature on e-service research [29, 59]. Indeed, Featherman and Pavlou [29] integrate perceived risk into the e-services adoption model and conclude that e-services adoption is adversely affected by performance-based risk perceptions and the customers' effort to adopt the e-service platform. Perception of technological risk prevents patients from using digital health platforms. For this reason, according to studies investigating the facilitated conditions to use of digital platforms in the healthcare industry [28], individual's control belief regarding the availability of resources and support structures to facilitate system use can affect the technology acceptance and the use of e-service positively [60, 61]. As a result, reduced cognitive effort and perceived risk levels should add to the IT system's instrumental benefits, such as increased performance [59].
- 3. Perceived ease of use. This dimension (which explains 5.78% of the variance of the phenomenon) is a determining aspect for a prolonged participation of the patient in telemonitoring activities. Patients initiate active and collaborative participation mechanisms only when the use of the platform is simple and understandable [36]. A perception of simplicity can guarantee prolonged use of the platform thus becoming a good substitute for the traditional health service. This factor constitutes an interesting indicator for the process of streamlining the work of doctors and customizing the service to the real needs of the patient.
- 4. Patient satisfaction: this dimension (which explains 9.57% of the variance of the phenomenon) measures the patient's response after the experience with the healthcare platform. It defines the patient's overall assessment and general impression. This aspect is essential for verifying the effectiveness of a "Connected Care" patient-centred approach with shared and integrated digital health models. According to the previous research [62, 63], to guarantee the best result, these models must be based on “relationship-centred care” that exploits patient engagement to guarantee a satisfactory result of the health service, especially in periods of overload of the health system. Previous studies demonstrated that satisfaction is influenced by the performance of e-services involving innovative and technological complex [64]. Moreover, recent literature demonstrates that satisfaction has a crucial role in e-services loyalty [65]. Therefore, because satisfaction is another critical factor activated into the digital platforms, it is crucial to have systems that guarantee continuous engagement contributing to patient and e-health service provider satisfaction with their relationships [62, 65].
- 5*. *Patient Cognitive Engagement: (explains 3.09% of the variance of the phenomenon) compared to what is stated in the literature on patient engagement which sees it as a three-dimensional construct (cognitive, emotional and behavioural) [6, 66], our research demonstrates that during the patient's interaction with the COVID-19 telemonitoring platform, cognitive engagement is a determining dimension in its own right that encourages the patient's cognitive engagement while using the platform. The results of this research highlighted that the patient's cognitive sphere is activated and engaged regardless of the emotional and behavioural dimension.
- 6. Perceived usefulness. This sixth dimension (which explains 2.67% of the variability of the phenomenon) includes all those aspects that justify the use, mostly prolonged (for 42% for more than a month, see Table 4), of the digital platform by patients. To ensure its prolonged use, the telemonitoring service must be perceived as effective and useful both for following the evolution of the infection and guaranteeing quick and easy feedback on the health status. This consideration is consistent with preceding research that demonstrates that telemonitoring services' effectiveness depends on perceived usefulness [67]. In particular, when a health digital platform does not guarantee feedback and patient involvement, it is not perceived as valuable and beneficial for patients [67]. On the other hand, both for patients and physicians, perceived usefulness positively affects the intention to use e-health services [59, 63, 67].

### Effectiveness of the Telemonitoring platform for COVID-19

Finally, on the basis of the results of the exploratory factor analysis, an ANOVA test was conducted to compare the different dimensions underlying the mechanisms of patient participation on the COVID-19 telemonitoring platform between the three different types of patients (infected, uninfected and with suspected SARS CoV-2 infection). This test helps to understand whether and which factors are different between the three types of patients. The results of the variance (Tables 5 and 6) obtained from the scores of the six factors highlighted a different perception of the level of satisfaction among the three groups of patients _MCOVIDYes_ = 0.173, SD = 1.18; M_COVID-free_ = -. 065, SD = 1.07; M_COVID-perhaps_ = -. 567, SD = 1.13).

**Table 5:** Differences between the types of patients and factors

Factors		Sum of Squares	df	Mean Square	F	Sig
Self-Health engagement	Between Groups	1.379	2	.690	.308	.736
	Within Groups	260.089	116	2.242		
	Total	261.468	118			
Technological risk	Between Groups	4.196	2	2.098	2.074	.130
	Within Groups	117.360	116	1.012		
	Total	121.556	118			
Perceived ease of use	Between Groups	4.598	2	2.299	1.726	.183
	Within Groups	154.513	116	1.332		
	Total	159.112	118			
Satisfaction	Between Groups	6.940	2	3.470	2.654	.075*
	Within Groups	151.658	116	1.307		
	Total	158.598	118			
Cognitive engagement	Between Groups	1.012	2	.506	.400	.671
	Within Groups	146.527	116	1.263		
	Total	147.538	118			
Perceived usefulness	Between Groups	4.016	2	2.008	1.184	.310
	Within Groups	196.772	116	1.696		
	Total	200.788	118			

* *p* value < 0.1

**Table 6:** Score for satisfaction according to patient type

Patient-target								
**Value**	**No COVID-19**		**Yes COVID-19**		**Perhaps COVID-19**		**Total**	
	N	%	N	%	N	%	N	%
** < 3**	5	50	5	50	0	0	10	100
** > 3 ≤ 5**	11	31.4	14	40.0	10	28.6	35	100
** > 5**	24	32.4	45	60.8	5	6.8	74	100
**Total**	40	33.6	64	53.8	15	12.6	119	100

In particular, as can be seen explicitly from Table 5 for patients infected with SARS CoV-2 (COVID_Yes_) there is a significant difference in the level of perceived satisfaction (t_COVIDYes_ (118) = 2.654; *p *= 0.075 *p* value =  < 0.1). While for all the other factors no differences were found between the two groups.

Then if we look at the levels of satisfaction for each group of patients, we can find significant differences. Table 6 shows the type of patient and their opinions on satisfaction. Out of the 74 patients who expressed satisfaction ratings higher than 5 on the 7-point Likert scale, more than 60% of the survey participants were among those who declared to have COVID-19. From the variance test and the differences between the groups by the level of satisfaction reached, we can show that the COVID-19 telemonitoring platform is effective for patient monitoring and that it activates self-health satisfaction. The satisfaction of COVID-19 patients more than the other patients can be explained by the perceived usefulness of the digital platform. Characteristics of the COVID-19 telemonitoring platform guarantees self-health management through patient involvement and physician interaction. The usefulness perceived by infected patients, the engagement, and possibility to interact with own physician has activated the satisfaction, that as demonstrated by previous research, is affected both by engagement [67] and the active roles in the management of their health in collaboration with health professionals [62, 64].

## Discussion

The COVID-19 pandemic has created a strong incentive to search for alternative solutions to the traditional systems of care and the provision of health services, and has found the answer to these new and pressing needs precisely in digital technology. The efficient national health system, with the adherence to health protocols to prevent COVID-19, could help to reduce the incidence of other infectious diseases [68] and the infection of children even if the prevalence, severity, and diversity of the symptoms are less than in adults [69]. Technology has proved to be capable of solving a series of problems aimed at avoiding the spread of the infection, the pressure on emergency rooms and the saturation of hospital beds. In particular, digital health engagement platforms are in all respects decisive telemedicine tools that can help the activation of health self-management systems and therefore facilitate remote monitoring systems, especially in times of pandemics. Such platforms work because they create an ecosystem in which users co-create value through collaboration and mutual participation [65, 70]. But despite the usefulness of digital tools in facilitating communication, monitoring pathologies and facilitating educational support, their diffusion has been slow and discontinuous [71]. The literature has highlighted the importance of engagement platforms activated by organizations through processes of co-creation of value between users, platform managers and stakeholders. Nevertheless, currently the role that digital health engagement platforms have on mechanisms activated for COVID-19 telemonitoring has not yet been fully investigated.

**Fig. 1:**
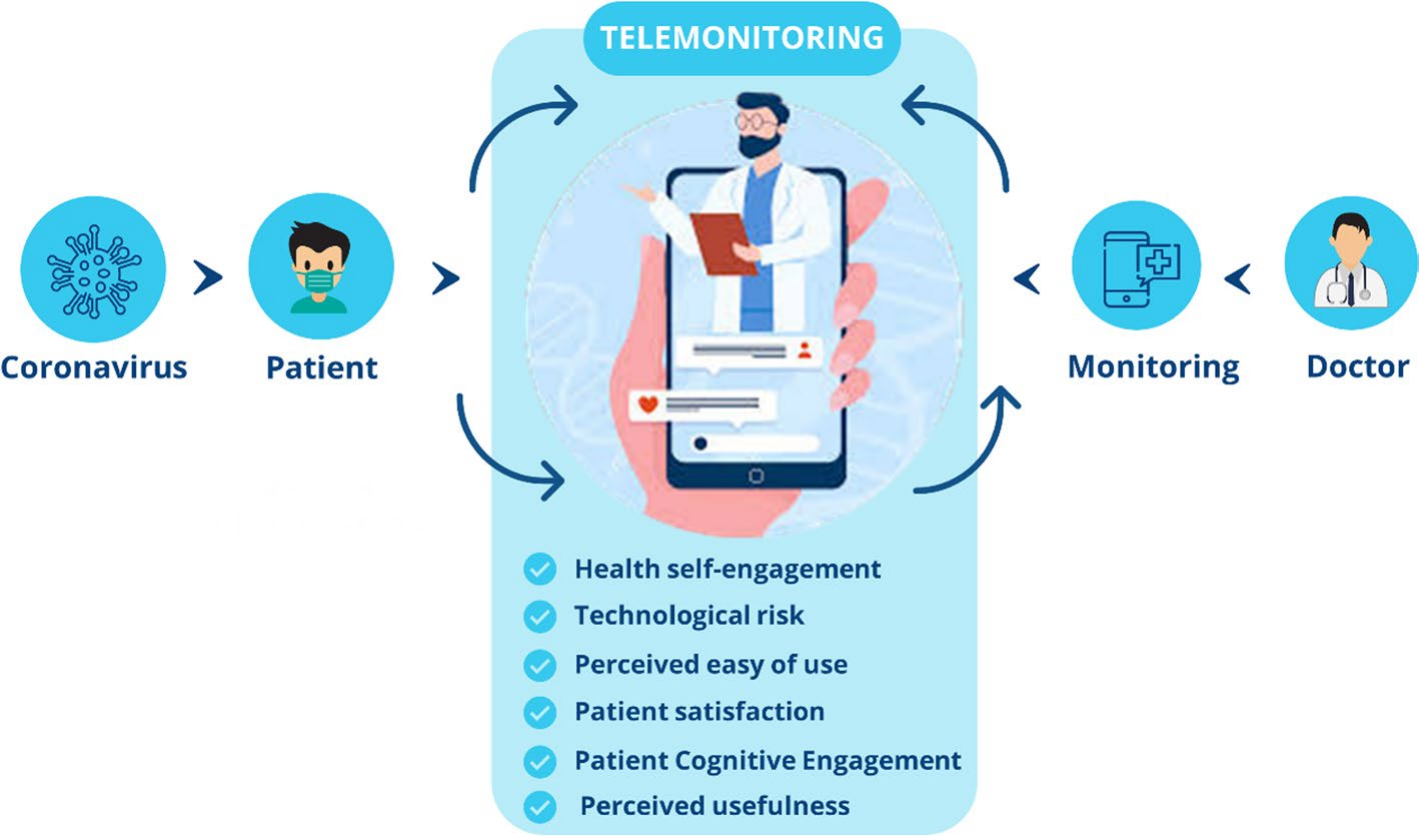
Key factors of digital health engagement platform for self-health management

This study aims at contributing to understanding the critical success factors of digital health tools (Fig. 1). Therefore, the paper starts from the main theoretical constructs involved in the use of telemonitoring platforms and goes through the evaluation of patient satisfaction and engagement in the use and acceptance of digital health platforms for COVID-19 telemonitoring. Source: our elaboration.

This empirical research confirms what the literature has already highlighted about the crucial role of engagement in medical telemonitoring platforms [5, 27, 65, 72], and how it positively impacts on self-health satisfaction [73, 74]. Acting as a lever for engagement, digital health platforms can in fact support the patient in the management of their state of health [62, 63, 65]. In addition, during a period of overload of the health system such as has just been the case due to the pandemic, home telemonitoring for COVID-19 can be a valuable aid for improving the efficiency and effectiveness of health systems [2, 10, 72]. During the different phases of the patient journey, it is therefore feasible to foresee increasingly tailor-made services. Such services can meet expectations and contribute to the evolution of the current health service by focusing on the patient and their psycho-physical well-being. Digital platforms represent a valid aid to self-health management, seeing as it is based on patient engagement; they reconcile the efficiency needs of the healthcare system with the benefits for patients [72]. Telemonitoring systems can lower public health intervention costs while increasing client engagement and service scalability. In addition, the convenience offered by the health digital platforms supporting the remote monitoring system likely contributed to patient satisfaction and high engagement levels. While technological risk, effectiveness, and easy use of the platform can be considered other crucial critical success factors for health-self management through digital platforms.

Furthermore, an additional contribution to this research highlights a new critical success factor in the use of telemonitoring platforms – the "Self-Health Engagement", capable of explaining patient engagement on COVID-19 telemonitoring platforms. In digital health contexts therefore, Self-Health Management passes firstly through cognitive engagement towards the digital platform, then to the perception of its usefulness and simplicity and a good predisposition to its use and finally, towards an engagement aimed at improving health status. These factors are critical to ensure the success of the platform and therefore the participation of the patient and the doctor in improving the state of health. This is consistent with Welch et al. (2017) [11], according to whom it is possible that patients will choose to use telemedicine if it is recommended by their general practitioner with whom they have a consolidated relationship. In close connection to this, further evidence in the paper highlights how the cognitive dimension of engagement assumes an autonomous role compared to the other dimensions (emotional and behavioural). This factor is particularly important since, as the literature confirms, a cognitive effort has a significant effect on the use of digital health platforms [75]. The results show that understanding the tool, confidence in its use and full knowledge of the platform's features can help ensure the continuity of telemonitoring services. The pandemic emergency has highlighted the crucial support of e-health services both in streamlining the work of physicians and offering a “tailor-made” health service related to the real need of patients. Since self-health engagement emerges as an unprecedented and relevant construct in the COVID-19 telemonitoring platform, public and private health service providers should ensure the quality of the service offered and active patient participation through feedback and perceptible, tangible benefits.

This paper confirmed the importance of engagement in health-self management and demonstrates the decisive role of digital technology and patient participation in self-management. Other authors have explored the factors affecting physician behavior based on the engagement dimensions activated by participation in the health digital platform [59, 65]. This paper explores the critical success factors in telemonitoring platforms during the COVID-19 emergency and finds the "health self-engagement" as an unreleased construct in the self-care research. In this way, this paper advances the study of patient engagement in the context of health e-services and enriches the literature on digital health care.

Finally, although significant differences in terms of engagement emerged between the patients involved in the survey in relation to their possible state of contagion (yes, no, perhaps), the contribution provided by this study to the understanding of self-management in digital health, as yet still not fully explored, suggests an effective diffusion of engagement platforms not only in conditions of health emergencies.

### Study limitation

This study provides an analysis of the main factors that determine patient participation on a COVID-19 telemonitoring platform. In order to explore these factors, we used the Italian platform of PagineMediche.it. We have explored the state of the art of only one country (Italy) failing to explore whether these factors are similarly activated in other countries affected by the COVID-19 pandemic. This latter aspect could be subject matter for further investigation taking into due consideration the cultural factors that could modify the results. In addition, the sample, obviously limited and therefore not representative, could be further extended in order to detect the effectiveness of telemonitoring platforms and their use for self-health management also after the pandemic. Despite these limitations, our study provides some indications on the mechanisms involved in the patient's interaction with a digital health monitoring platform, including self-health engagement which assumes unprecedented connotations in the literature on patient engagement in the context of e-health.

## Conclusions

In conclusion, our study demonstrates that COVID-19 telemonitoring platforms are effective healthcare engagement platforms. Engagement is the crucial element for the use of these platforms. They engage the patient both on the emotional-behavioural and on the cognitive profile. These platforms are also satisfactory for patient monitoring, thus facilitating the doctors’ tasks during the remote assistance of their patients. For healthcare platform service providers, it could prove to be very useful to identify and understand the critical success factors of these platforms, especially to ensure patient satisfaction and optimize their effectiveness. Platforms capable of clearly guaranteeing the monitoring of the patient's state of health and where improvement is clearly perceived are favoured over one-way and mainly doctor-orientated ones. At the same time, governments, engaged in the ongoing health emergency, can verify the effectiveness of this method of health treatment and make informed decisions on how to direct health expenditure towards services that are truly closer to the real needs of the patient [59, 62–64, 67, 72–74].

## Supplementary Information

**Additional file 1.** 

**Additional file 2. **

## Data Availability

The datasets used and analysed during the current study are available from the corresponding author on reasonable request.
