# Supplementary material for: Key drivers involved in the telemonitoring of covid-19 for self-health management: an exploratory factor analysis
Source: BMC Health Serv Res. 2022 Apr 19;22:520. doi: 10.1186/s12913-022-07828-3 (PMC9016691; doi:10.1186/s12913-022-07828-3)
Supplement: Supplementary file 1 — Additional file 1. [file 12913_2022_7828_MOESM1_ESM.pdf]

DocuSign Envelope ID: 0B0CA72A-18B8-47CC-AF81-9383B0A45527

paginemediche

Salerno (Italy)
28th February 2022

To whom it may concerns:

With reference to the study conducted by Lo Presti L., Testa M., Maggioni G. and Marino V., entitled "Key Drivers Involved in the Telemonitoring of Covid-19 for Self-Health Management: an Exploratory Factor Analysis", we confirm that the survey included had been performed in accordance with the anonymity and total respect for privacy has been granted pursuant to Regulation (EU) no. 2016/679 - European Union regulation on the processing of personal data and privacy.

Customers have been informed that the data collected would have been processed in compliance with the security measures required by law and the information provided would have been used exclusively for research purposes and disseminated externally only in aggregate form.

Since the survey was conducted on clients of a payment health service platform and no clinical trials were conducted, the Board of Paginemediche.it believes that the approval procedure of the Ethics committee is not needed, and it is not applicable for this research. Those who agreed to participate with informed consent were surveyed. Participant's privacy and confidentiality were protected.

Sincerely,

Institutional Review Board
Graziella Bilotta (Chief Executive Officer)
Alessandro Sbenaglia (Chief Technology Officer)
Emanuele Urbani (Chief Medical Officer)

Graziella Bilotta

Graziella Bilotta
