# Supplementary material for: Key drivers involved in the telemonitoring of covid-19 for self-health management: an exploratory factor analysis
Source: BMC Health Serv Res. 2022 Apr 19;22:520. doi: 10.1186/s12913-022-07828-3 (PMC9016691; doi:10.1186/s12913-022-07828-3)
Supplement: Supplementary file 2 — Additional file 2. [file 12913_2022_7828_MOESM2_ESM.docx]

**Appendix A.**

| **N.** | **Factor** | **Items** | **Code** |
| --- | --- | --- | --- |
| 1 | Health self-engagement | The use of telemonitoring has increased the effectiveness of health treatment. | PB1 |
|  |  | I feel good when I use the telemonitoring service. | ENGe2 |
|  |  | The use of telemonitoring has increased my awareness of my ailments. | PB3 |
|  |  | I feel reassured when I use the telemonitoring service. | ENGe3 |
|  |  | Using telemedicine has increased my faith in the therapy. | PB4 |
|  |  | Thanks to telemonitoring I have increased the frequency with which I monitor my health. | ENGb2 |
|  |  | I prefer to use telemonitoring rather than the traditional health service. | ENGb3 |
|  |  | The use of telemonitoring has increased my psychic-physical well-being. | PB2 |
|  |  | Telemonitoring helps me change the way I manage my ailments. | ENGb1 |
| 2 | Technological risk |  |  |
|  |  | The use of telemonitoring could cause problems in safeguarding my privacy. | EE2 |
|  |  | There are security problems linked to the use of telemonitoring services. | PTR2 |
|  |  | With telemonitoring services there is a risk of leakage of personal information. | PTR1 |
|  |  | The use of telemedicine could interfere with my habits. | EE4 |
|  |  | The use of telemonitoring services could have negative consequences. | PTR3 |
|  |  | Screen size, internet velocity etc. Cause problems for the use of telemonitoring services. | EE3 |
|  |  | Adapting to the telemonitoring service requires great effort. | EE1 |
| 3 | Perceived ease of use |  |  |
|  |  | The use of the telemonitoring service is clear and understandable. | PEU2 |
|  |  | It is easy to understand how to use it. | PEU1 |
|  |  | The telemonitoring service is easy to use. | PEU4 |
|  |  | The use of the telemonitoring service is flexible. | PEU3 |
| 4 | Satisfaction |  |  |
|  |  | I am satisfied with what the telemonitoring services offers me. | SAT2 |
|  |  | This telemonitoring services meets my expectations. | SAT1 |
|  |  | If I could go back in time I would confirm my adhesion to the telemonitoring service. | SAT3 |
| 5 | Patient cognitive engagement |  |  |
|  |  | I would like to try other techniques of telemonitoring. | ENGc2 |
|  |  | I would like to know more about telemedicine. | ENGc1 |
|  |  | I would like to be informed on the next telemonitoring initiatives. | ENGc3 |
| 6 | Perceived usefulness |  |  |
|  |  | I find it easy to obtain and verify information on my state of health through the telemonitoring service. | PU2 |
|  |  | With telemonitoring I can quickly access information on my health status. | PU1 |
|  |  | The telemonitoring service is effective. | PU3 |
|  |  | The telemonitoring service is useful. | PU4 |
